# Expected long-term impact of screening endoscopy on colorectal cancer incidence: a modelling study

**DOI:** 10.18632/oncotarget.10178

**Published:** 2016-06-20

**Authors:** Hermann Brenner, Jens Kretschmann, Christian Stock, Michael Hoffmeister

**Affiliations:** ^1^ Division of Clinical Epidemiology and Aging Research, German Cancer Research Center (DKFZ), Heidelberg, Germany; ^2^ Division of Preventive Oncology, German Cancer Research Center (DKFZ) and National Center for Tumor Diseases (NCT), Heidelberg, Germany; ^3^ German Cancer Consortium (DKTK), German Cancer Research Center (DKFZ), Heidelberg, Germany; ^4^ Central Research Institute of Ambulatory Health Care in Germany, Berlin, Germany; ^5^ Institute of Medical Biometry and Informatics, University of Heidelberg, Heidelberg, Germany

**Keywords:** cohort studies, colorectal cancer, screening, trials

## Abstract

**BACKGROUND & AIMS:**

Screening endoscopy reduces colorectal cancer (CRC) incidence but the time course and magnitude of effects beyond 10 years after screening are unknown. We aimed to estimate the expected time course and magnitude of long-term impact of screening endoscopy on CRC incidence.

**METHODS:**

We used Markov models based on the natural history of the disease along with data from the German national screening colonoscopy registry to derive the expected impact of screening colonoscopy at age 55 or 60 on cumulative CRC incidence according to time of follow-up over a period of up to 25 years.

**RESULTS:**

After a single screening colonoscopy, cumulative CRC incidence is expected to be increased for approximately 4 to 5 years. This transient increase is expected to be followed by a steadily increasing reduction in cumulative CRC incidence for at least 25 years. Less than one third of this long-term reduction is expected to be seen within 10-12 years of follow-up, the length of follow-up reported on in RCTs on flexible sigmoidoscopy screening and in most cohort studies on both sigmoidoscopy and colonoscopy screening. In relative terms, risk reduction is expected to reach its maximum approximately 15 years after a single screening colonoscopy and 20-25 years after the initial screening colonoscopy in case of repeat screening colonoscopy after 10 years.

**CONCLUSIONS:**

The long-term impact of screening endoscopy on CRC prevention is expected to be much stronger than suggested by currently available evidence from RCTs and cohort studies with limited length of follow-up.

## INTRODUCTION

Several randomized clinical trials (RCTs) have demonstrated effective reduction of colorectal cancer (CRC) incidence by screening with flexible sigmoidoscopy (FS). [1–5] Observational studies likewise suggest strong reduction of CRC incidence by screening FS and screening colonoscopy. [5] Results from RCTs on the impact of screening colonoscopy on CRC incidence are not available yet. An RCT addressing this issue has completed recruitment only very recently, with main results expected after 15 years of follow-up only. [6]

A crucial issue in reporting and interpreting the results of studies on the impact of screening FS or colonoscopy on CRC incidence is the length of follow-up: Detection of prevalent CRC cases at screening leads to an apparent initial increase in CRC incidence, which is typically followed by very low incidence in subsequent years. As a result, observed net effects on cumulative CRC incidence are rapidly changing with length of follow-up. For example, in the screening FS RCTs, cumulative CRC incidence in the intervention group consistently exceeded CRC incidence in the control group until approximately four years after recruitment, whereas substantial reduction of cumulative incidence was observed after median follow-up times of 10.5 to 11.9 years. [1–4] None of the FS trials has so far reported results for longer follow-up times, and no results at all on cumulative CRC incidence are available so far from the aforementioned screening colonoscopy trial. With few exceptions, [7,8] observational studies were typically also restricted to no more than 12 years of follow-up after screening FS or colonoscopy. [5] There is therefore very limited direct evidence, which effects of screening endoscopy are to be expected in the longer run. Knowledge of the expected time course of effects would though be of utmost interest for planning and interpreting screening endoscopy studies and for implementing and evaluating screening endoscopy programs. In this article, we use Markov models based on the natural history of the disease to derive the expected time course of screening colonoscopy effects over a follow-up period of up to 25 years.

## MATERIALS AND METHODS

### Database

Our analysis is based on data from the German screening colonoscopy program. Screening colonoscopy has been offered in Germany as a primary screening examination for early detection and prevention of CRC since October 2002. Women and men are entitled to have a first screening colonoscopy at age 55 or older. If this first screening colonoscopy is conducted before 65 years of age, a second screening colonoscopy is offered 10 years later.

Along with introduction of screening colonoscopy, a national screening colonoscopy registry was built up. Details of its operation have been reported elsewhere. [9] Briefly, all screening colonoscopies are reported anonymously on a standardized form. Reporting is virtually complete, as it is a prerequisite for physicians’ reimbursement by the health insurance funds. The registry includes only primary screening examinations (i.e., colonoscopies conducted for surveillance, work-up of symptoms or other screening tests are not included). Items reported include, besides basic sociodemographic variables, findings at colonoscopy, including number, size and histological characteristics of polyps. In case of multiple neoplasms, only the most advanced one (nonadvanced adenoma, advanced adenoma, or cancer) is recorded. Advanced adenomas are defined as at least 1 adenoma ≥ 1 cm or at least 1 adenoma with villous components or high-grade dysplasia.

Approximately 20-30% of eligible people had a screening colonoscopy within the initial 10 years from the introduction of this screening offer. For this analysis, we used data from 344,658 and 207,244 first time participants of screening colonoscopy in 2003-2012 aged 55 and 60 years, respectively, as a basis for simulating the expected impact of screening colonoscopy conducted at those ages.

### Statistical analysis

**Figure 1:**
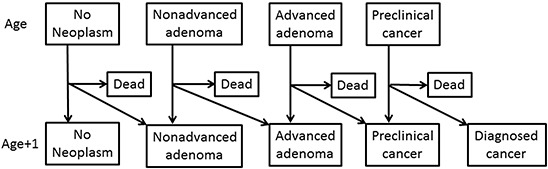
Schematic presentation of the Markov model used for the analyses

We first determined sex specific prevalences of the most advanced finding (no neoplasm, nonadvanced adenoma, advanced adenoma, preclinical CRC) at screening colonoscopy among men and women undergoing screening colonoscopy at age 55 (Table 1). We used 5 state Markov models implemented in Excel spreadsheets with states representing the most advanced colorectal lesion (no neoplasm, nonadvanced neoplasm, advanced neoplasm, preclinical CRC, diagnosed CRC) and annual iterations as previously described [10] and outlined in Figure 1. At each iteration, progression between states was modelled based on previously derived sex and age specific annual transition rates (Table 2), [11–13] accounting for mortality which was obtained from general population life tables for Germany in the period 2009/2011. [14]

**Table 1:** Prevalences of most advanced findings among 55- and 60-year old male and female participants of screening colonoscopy. German screening colonoscopy registry, 2003-2012

Sex	Age	Most advanced finding at colonoscopy								Total	
		No neoplasm		Nonadvanced adenoma		Advanced adenoma		Cancer			
		n	%	n	%	n	%	n	%	n	%
Men	55	110,521	77.3	23,315	16.3	8,490	5.9	656	0.46	142,982	100
	60	67,635	72.9	17,037	18.4	7,323	7.9	792	0.85	92,787	100
Women	55	174,774	86.7	19,894	9.9	6,501	3.2	507	0.25	201,676	100
	60	95,930	83.8	13,006	11.4	5,010	4.4	511	0.45	114,457	100

**Table 2:** Sex and age specific transition rates (summary and synopsis of estimates derived in references 10-12)

Sex	Age	Annual transition rates in %							
		No neoplasm ↓ nonadvanced adenoma		Non-advanced adenoma ↓ advanced adenoma		Advanced adenoma ↓ preclinical CRC		Preclinical CRC ↓ clinical CRC	
		PE	95% CI	PE	95% CI	PE	95% CI	PE	95% CI
Men	55-59	2.4	2.2-2.6	4.2	3.8-4.6	2.6	2.4-2.9	18.1	16.7-19.5
	60-64	2.3	2.1-2.6	4.0	3.6-4.4	3.1	2.8-3.3	19.2	18.1-20.3
	65-69	2.4	2.1-2.6	4.0	3.6-4.3	3.8	3.5-4.1	21.3	20.3-22.4
	70-74	2.2	1.8-2.5	4.1	3.6-4.6	5.1	4.8-5.5	20.6	19.5-21.7
	75-79	1.8	1.2-2.3	3.7	2.9-4.6	5.2	4.6-5.8	20.1	18.9-21.4
Women	55-59	1.4	1.3-1.5	4.0	3.6-4.5	2.5	2.2-2.7	21.3	19.5-23.4
	60-64	1.5	1.4-1.7	3.6	3.2-4.1	2.7	2.4-3.0	22.5	20.9-24.2
	65-69	1.6	1.4-1.8	3.7	3.2-4.1	3.8	3.5-4.1	21.9	20.6-23.3
	70-74	1.6	1.3-1.8	4.7	4.1-5.3	5.0	4.5-5.4	20.8	19.4-22.2
	75-79	1.2	0.8-1.6	3.7	2.8-4.7	5.6	4.9-6.3	19.2	17.9-20.7

CI, confidence interval; CRC, colorectal cancer; PE, point estimate

Markov models were employed starting at age 55 and running up to a maximum age of 80 to assess the expected cumulative incidence of CRC between age 55 and each single year of age from 56 to 80 years in the absence and in the presence of screening colonoscopy. Expected cumulative incidences in the absence of screening were derived by starting with age and sex specific prevalences of the various states as shown in Table 1 and applying the transition rates between various states listed in Table 2, assuming that no intervention was made. Expected cumulative incidences after a single screening colonoscopy at age 55 was modelled in such a way (discussed in detail below) that participants with preclinical CRC were immediately moved to the category “diagnosed CRC” due to detection of their cancer at screening colonoscopy, whereas participants carrying adenomas were set back to the category “no neoplasm”, assuming that their adenomas were removed at screening colonoscopy. Modelling was then continued in subsequent years starting from the updated prevalences and assuming the annual transition rates shown in Table 2.

Expected cumulative incidence of CRC for follow-up periods from 1 to 25 years (i.e., up to ages 56 to 80 years) was determined and compared between populations with and without screening. The expected impact of screening on cumulative incidence of CRC was expressed both in relative and absolute terms by the ratio and difference of the expected cumulative incidence of CRC of screened and unscreened individuals, respectively. For simplicity, this ratio and difference are denoted relative risk and risk difference thereafter.

Analogous calculations were done for assessing the expected impact of a single screening colonoscopy at age 60 and follow-up periods of 1 to 20 years (i.e., up to ages 61 to 80). Finally, analogous calculations were done for assessing the expected impact for follow-up periods of 1 to 25 years after an initial screening colonoscopy at age 55 and a repeat screening colonoscopy at age 65. In models assuming repeat screening colonoscopies, participants with preclinical CRC were again moved to the category “diagnosed CRC” and participants with adenomas were again moved to the category “no neoplasm” during the year of the repeat screening colonoscopy.

To account for uncertainties in the transition rates shown in Table 2, sensitivity analyses were conducted in which all point estimates of transition rates were replaced by either the lower or the upper end of their confidence intervals.

## RESULTS

Table 1 shows the prevalences of various types of colorectal neoplasms among 55 and 60 year old participants of screening colonoscopy in Germany between 2003 and 2012. Prevalences of nonadvanced adenomas, advanced adenomas and cancer were 16.3%, 5.9% and 0.46%, respectively among 55 year old men. Prevalences were 40-46% lower among 55 year old women. Among both men and women, slightly higher prevalences were observed at age 60.

**Figure 2:**
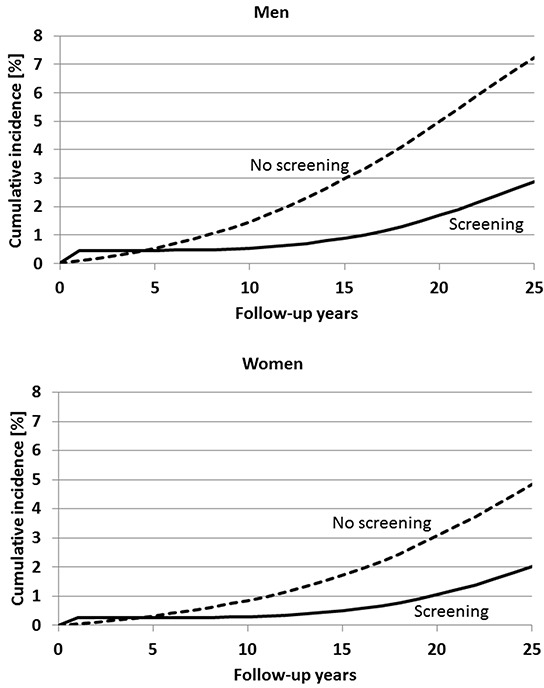
Expected cumulative incidence of colorectal cancer within 25 years after a single screening colonoscopy at age 55 compared to no screening

Figure 2 shows the expected cumulative incidence of CRC after a single screening colonoscopy at age 55 compared to no screening. Among men, cumulative incidence is expected to increase to approximately 7% after 25 years (i.e., at age 80) in the absence of screening. Detection of preclinical cancers at screening colonoscopy at age 55 is expected to lead to a transiently increased cumulative incidence for approximately 4 to 5 years, but a substantially reduced cumulative incidence is expected in later years, reaching approximately 3% after 25 years. Similar patterns are expected among women. However, overall levels of cumulative incidence are expected to be lower among women, reaching approximately 5% and 2% within the 25-year period from age 55 to 80 in the absence and presence of screening colonoscopy at age 55, respectively.

**Figure 3:**
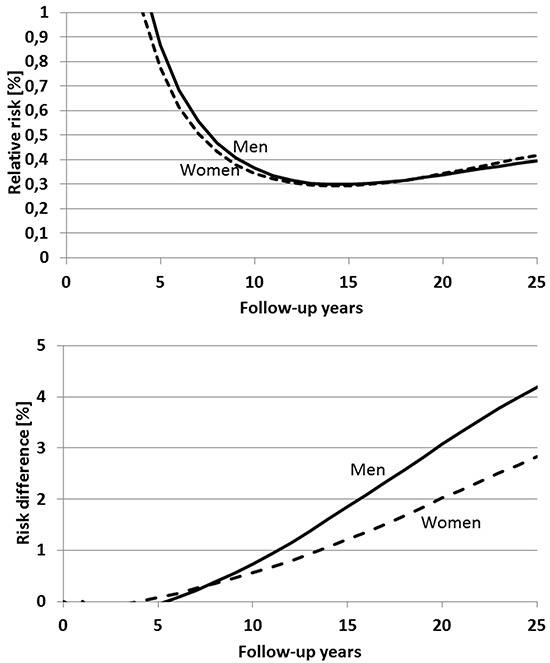
Expected time course of relative risk (upper panel) and risk difference (lower panel) of colorectal cancer after a single screening colonoscopy at age 55 compared to no screening

Figure 3, upper panel, shows the expected relative risk in men and women with a single screening colonoscopy at 55 years of age compared to men and women without screening by year of follow-up from age 55 on. In both men and women, relative risk among screened participants is expected to decrease from levels above 1 up to the 5^th^ year of follow-up to levels around 0.35 after 10 years of follow-up. A further decrease of relative risk to minimum levels around 0.3 is expected after approximately 15 years of follow-up. For longer periods of follow-up, the relative reduction of risk is expected to be slightly less pronounced, with relative risks around 0.4 expected after 25 years of follow-up.

By contrast, absolute reduction of cumulative incidence is expected to steadily increase at a relatively constant rate over the entire 25-year follow-up period, reaching 4.4 and 2.8 percent units among men and women, respectively, after 25 years (Figure 3, lower panel). Ten to twelve years after screening colonoscopy, less than one third of this risk reduction would be expected to be seen.

**Figure 4:**
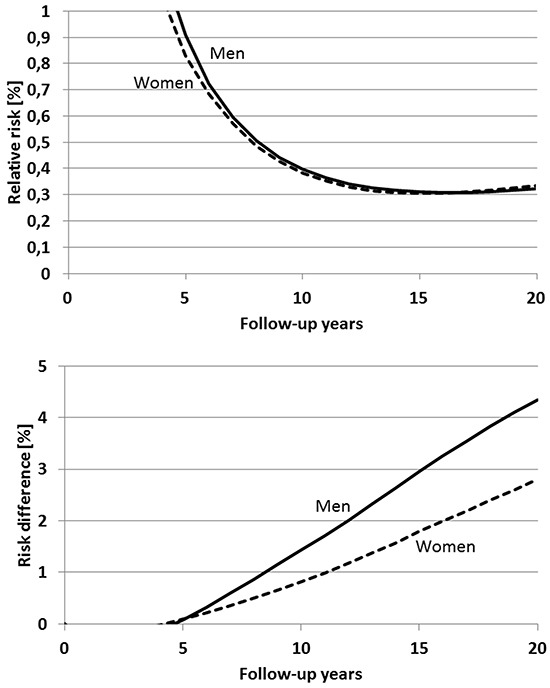
Expected time course of relative risk (upper panel) and risk difference (lower panel) of colorectal cancer after a single screening colonoscopy at age 60 compared to no screening

Overall, very similar patterns of risk reduction after an apparent initial increase are expected after a single screening colonoscopy at age 60 (Figure 4). Relative risk is likewise expected to reach a minimum close to 0.3 after approximately 15 years, whereas risk reduction in absolute terms is expected to steadily increase to levels of 4.3 and 2.8 percent units among men and women, respectively, after 20 years, i.e., at age 80 of the screened cohort.

**Figure 5:**
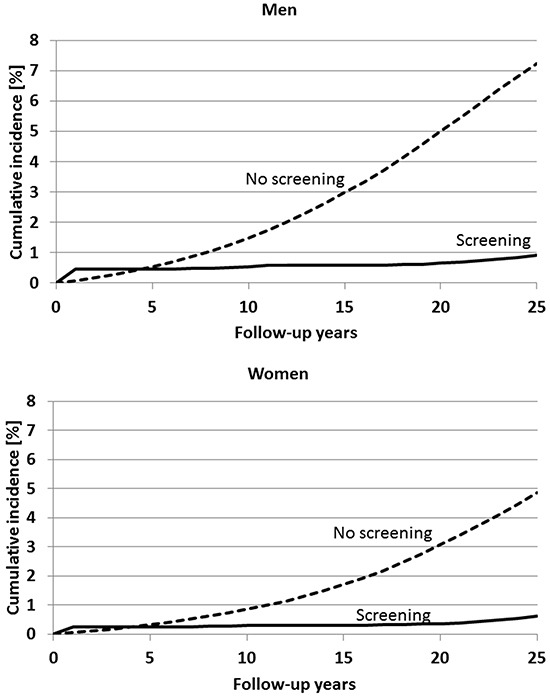
Expected cumulative incidence of colorectal cancer within 25 years cancer in case of a first and second screening colonoscopy at ages 55 and 65, respectively, compared to no screening Follow-up time refers to time since first screening.

**Figure 6:**
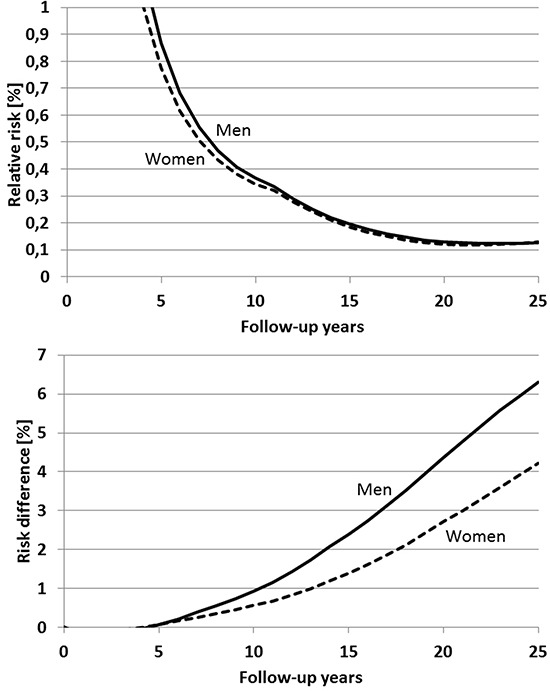
Expected time course of relative risk (upper panel) and risk difference (lower panel) of colorectal cancer in case of a first and second screening colonoscopy at ages 55 and 65, respectively, compared to no screening Follow-up time refers to time since first screening.

A second screening colonoscopy at age 65 after first screening colonoscopy at age 55 would be expected to substantially enhance the screening effect: 25-year cumulative incidence would be expected to remain below 1% in both men and women (Figure 5). Relative risk compared to no screening would be expected to steadily decrease for at least 20 years to levels slightly above 0.1 in both men and women (Figure 6, upper panel), and absolute risk reduction would be expected to steadily increase up to 6.3 and 4.2 percent units among men and women, respectively, 25 years after the first screening colonoscopy (Figure 6, lower panel).

Sensitivity analyses using lower or upper ends of the confidence intervals of the transition rates shown in Table 2 yielded slightly lower or higher cumulative incidences and risk differences, respectively, but the time course of the changes in these parameters and both the absolute values and the time course of all relative risks were virtually identical. In particular, minimum values of relative risks of 0.3 approximately 15 years after a single screening colonoscopy at ages 55 or 60, and slightly above 0.1 approximately 20-25 years after repeat screening colonoscopies at ages 55 and 65 were seen in all scenarios.

## DISCUSSION

In this study, we derived the expected time course and expected magnitude of the effects of screening colonoscopy on CRC incidence. We performed model calculations based on findings at screening colonoscopy from the German national screening colonoscopy registry and previously derived parameters of the natural history of CRC development. Our results suggest relative risk to reach a minimum of approximately 0.3 approximately 15 years after a single screening colonoscopy at age 55 and a further decrease to slightly more than 0.1 as late as 20-25 years after the initial screening colonoscopy in case of a repeat screening colonoscopy at age 65. Absolute reduction of CRC risk is expected to steadily and strongly increase throughout the 25 year follow-up period considered in this analysis even after a single (once only) screening colonoscopy. Ten to twelve years after screening colonoscopy, less than one third of this risk reduction is expected to be seen. These patterns were remarkably robust against variation in transition rates in sensitivity analyses.

Obviously, the validity of our results depends on the validity of the underlying model assumptions. Most detailed and precise estimates of key parameters, such as sex and age specific prevalences of various precursor states of CRC and transition rates between them were derived from the German screening colonoscopy registry, the world's largest registry of its kind. [9,11–13] Although there is no gold standard against which to validate the model parameters, comparison of the magnitude and time course of modelled effects with effects observed in RCTs and cohort studies may provide clues as to the plausibility and validity of our modelling. As RCT evidence is not available for colonoscopy yet, we will focus our comparison to the time course of effects on distal CRC incidence in the FS trials and total CRC incidence in observational studies on screening colonoscopy.

Our finding that cumulative incidence is expected to be higher in the screening group than in the unscreened group for approximately 4 to 5 years and lower thereafter closely matches observations from the FS trials where curves of cumulative incidence of both groups consistently crossed after 3 to 5 years of follow-up. [1–4] Likewise, the expected strong time dependency of relative risk estimates within the initial 10 years of follow-up closely matches observations from the Norwegian trial. In this RCT, no effect of screening FS on distal CRC incidence had initially been observed after a median follow-up of 7 years. [15] A clear and statistically significant effect was later reported after a median follow-up of 10.9 years. [4] None of the screening FS trials so far reported results for follow-up times beyond 12 years.

We modelled effects comparing people actually having screening colonoscopy and people not undergoing CRC screening. Our results should therefore be compared to per-protocol rather than intention-to-screen estimates of RCTs. The British and the Italian FS screening RCTs reported per-protocol relative risks of distal CRC of 0.50 and 0.60 after 11.2 and 10.5 years of follow-up, respectively. These results reflect somewhat less pronounced risk reductions than the relative risks of around 0.35 expected after this length of follow-up according to our models. One possible explanation for this apparent difference could be some contamination of the control groups in the RCTs, i.e., conduction of endoscopy in some proportion of the control group. [16] Our modelled relative risk estimate between 0.3 and 0.4 after approximately 10 or more years of follow-up closely matches the summary relative risk of 0.31 recently estimated in a meta-analysis of observational studies comparing people with and without screening colonoscopy. [5]

Another indirect check of the plausibility of our model results is the comparison of modelled cumulative incidence of CRC with cumulative CRC incidence derived from cancer registry data. Using cancer registry data from 2003, [17] the first year of screening colonoscopy in Germany, and accounting for general mortality in the same way as in our model calculations, cumulative incidence of CRC from age 55 to 80 would be estimated as 5.4% and 3.6% among men and women, respectively. These estimates are approximately 25% lower than the estimates derived for the unscreened population from our models. A possible explanation for this apparent difference could be that a non-negligible proportion of older adults in Germany have diagnostic colonoscopy at some time between 55 and 80 years of age [18–20] which also has strong protective effects by removal of adenomas [20].

In our models participants of screening colonoscopies in whom adenomas were detected were set back in the category “no adenoma” because adenomas are typically removed once detected. An implicit assumption of this approach is that these people subsequently have the same risk of developing CRC as people of the same sex and age with no adenomas. This assumption is likely to be violated to some extent because presence of adenomas is an indicator of increased risk (e.g., through risk factors, such as family history, smoking, red meat consumption etc. [21–23]). Such increased risk is expected to prevail even after removal of adenomas. [24] Also increased risk may prevail if not all adenomas are removed. On the other hand, surveillance colonoscopies that are recommended after detection and removal of adenomas might partly or fully compensate for this excess risk. [25–26] Depending on the extent of such compensation, which also depends on organization of and adherence to surveillance, [27] net risk reduction might be somewhat weaker or stronger than suggested by our models.

Our study has specific strengths and limitations. Strengths include reliance and derivation of key data and parameters on a very large national database of screening colonoscopies. A limitation is that this database includes data for screening colonoscopies from age 55 on only, the age from which screening colonoscopy is offered in Germany. Therefore, analyses could not be performed in an analogous manner for screening colonoscopies conducted at age 50, the age for initiation of screening colonoscopy in the average risk population often recommended by expert panels. [e.g. 25,26,28]

Our modeling was restricted to reduction of CRC incidence. We did not expand our models to assessment of reduction in CRC mortality. Such extension would have required additional assumptions on critical and rather uncertain parameters, such as sex and age specific survival after screening detected and clinically detected CRC. In contrast to CRC incidence, no initial increase after screening is expected for CRC mortality. Beneficial screening effects of CRC screening on CRC mortality might therefore be expected to manifest earlier, and they might furthermore be larger due to enhanced chances of cure of screening detected CRC cases. On the other hand, some delay in manifestation of beneficial screening effects on CRC mortality would be expected from the survival time after diagnosis. Apart from the differences in the initial years after screening, rather similar magnitude and time course of effects have been observed for CRC incidence and mortality in the screening FS trials. [1–4]

Given the lack of sufficiently reliable site specific transition rates, we were unable to provide subsite specific analyses, despite the likely major differences in effectiveness of screening colonoscopy for prevention of proximal and distal CRC. [29,30] Finally, our analysis exclusively considered CRC developing through the adenoma-carcinoma sequence. Although the vast majority of CRC is thought to develop this way, screening colonoscopy would be expected to have much smaller effects if any on the minority of other CRC. Therefore, the impact of screening colonoscopy on overall CRC incidence would be expected to be slightly smaller than estimated in our analyses.

Obviously, our modelling study only considered removal of adenomas actually detected at screening colonoscopy. Although non-negligible proportions of very small adenomas may be missed at colonoscopy, only a very small proportion of those adenomas would develop into clinically manifest cancer even during the 20-25 year follow-up periods considered in our analysis. Vice versa, although such development may occur in a non-negligible proportion of large adenomas, the proportion of missed large adenomas is expected to be very low. Therefore, the potential impact of missed adenomas is likely to be very small.

Despite the limitations, our results have important implications for the design and interpretation of RCTs and cohort studies aimed to assess the impact of screening colonoscopy, and similar implications are likely to apply for studies on the impact of screening FS (with distal rather than total CRC incidence as main outcome). In particular, our results demonstrate that any effect measures are expected to be strongly dependent on follow-up time. Meaningful relative risk estimates can only be expected beyond 10 years of follow-up. Absolute risk reduction is expected to steadily and strongly increase over decades, with only a minor share expected to be seen after 10 to 12 years, the range of median follow-up of available reports on screening FS trials. Long-term absolute effects of screening endoscopy are expected to be much stronger than suggested by existing evidence.
